# Transit-Amplifying Cells in the Fast Lane from Stem Cells towards Differentiation

**DOI:** 10.1155/2017/7602951

**Published:** 2017-08-01

**Authors:** Emma Rangel-Huerta, Ernesto Maldonado

**Affiliations:** EvoDevo Lab, Unidad de Sistemas Arrecifales, Instituto de Ciencias del Mar y Limnología, Universidad Nacional Autónoma de México, Puerto Morelos, QROO, Mexico

## Abstract

Stem cells have a high potential to impact regenerative medicine. However, stem cells in adult tissues often proliferate at very slow rates. During development, stem cells may change first to a pluripotent and highly proliferative state, known as transit-amplifying cells. Recent advances in the identification and isolation of these undifferentiated and fast-dividing cells could bring new alternatives for cell-based transplants. The skin epidermis has been the target of necessary research about transit-amplifying cells; this work has mainly been performed in mammalian cells, but further work is being pursued in other vertebrate models, such as zebrafish. In this review, we present some insights about the molecular repertoire regulating the transition from stem cells to transit-amplifying cells or playing a role in the transitioning to fully differentiated cells, including gene expression profiles, cell cycle regulation, and cellular asymmetrical events. We also discuss the potential use of this knowledge in effective progenitor cell-based transplants in the treatment of skin injuries and chronic disease.

## 1. Introduction

Stem cells (SCs) possess the capacity to self-renew and at the same time to differentiate into specialized cell types. This process is essential during development to form new tissues and organs and during adulthood to replenish cellular masses or to repair damaged organs. It is an evolutionarily conserved trait in animals, and there is evidence that this process is present in Cnidarians (like hydra) [1], Sponges [2], and Ctenophores (also known as comb jellies) [3], organisms located at the base of the animal phylogenetic tree. Therefore, mechanisms regulating cell proliferation and directing the fate of SC progenitors are highly conserved [4]. It is believed that, at some point, all basal animals had adult pluripotent cells (called primordial stem cells (PriSCs)) with the ability to function as SCs or as germ cells.

One of the challenges of cell transplant-based therapies is to induce SCs to proliferate and differentiate when needed. Therefore, it is essential to identify SC genes that can activate cell division and differentiation programs, considering that while many of these genes will be shared among SCs from diverse tissues, some others will be different or will be activated at various moments. Since some SCs from adult tissue remain almost quiescent, without dividing for long periods of time, it is important to study how cell proliferation is activated and terminated. Furthermore, controlling the balance between self-renewal and differentiation requires a fine tuning in different cell functions, such as chromatin remodeling, transcription, posttranscriptional modifications and translation [5–7]. These complex processes are regulated by multiple genetic pathways acting at different levels of regulation.

A logical path in understanding how SCs work is to identify and compare the set of genes that are expressed in SC progenitors with those active in the differentiated cells they produce; however, there is another level of complexity to consider. When SCs proliferate, they divide asymmetrically generating one SC and one cell committed to differentiation; however, it has been thoroughly documented that in many tissues and organs, SCs divide into one SC and one pluripotent transit-amplifying cell (TAC). TACs proliferate rapidly, and after several rounds of cell division, they become differentiated [8]. The essential feature of this “transit” cell population, as suggested by Loeffler and Potten [9], is their capacity to generate many maturing cells from very few cells. The cells entering the transit stage, or TACs, are capable of rapidly producing many differentiated cells, not only during development but also during regeneration.

One of the main problems in cell transplant-based therapies is the limited use of adult stem cells since these cells tend to remain almost quiescent, without dividing for long periods of time. Therefore, it is important to understand how SC progenitors are triggered to proliferate and differentiate rapidly, implying that any knowledge about TAC biology could be essential for designing new therapies. Here, we review some key aspects of TACs' characteristics and functions, with an emphasis on studies in epidermal skin cells from different organisms. First, we describe how the concept of TACs was shaped and their characteristics in cell proliferation and gene expression compared with SCs; we then present key aspects in the transition from SCs to TACs and later to differentiated cells. Finally, we summarize some information about the potential use of SCs and TACs in cell-based transplants to treat skin injuries and chronic disease.

## 2. Stem Cells and Transit-Amplifying Cells

Self-renewal and the capacity to differentiate into specific cells are the defining properties of SCs, as established early by McCulloch and Till in 1961, based on their experiments on “spleen colony-forming units” from bone marrow [7, 10]. At the same time, they established that SCs possess unlimited proliferative potential and pluripotency; however, in steady state conditions, SCs behave as slow proliferating cells [7]. In one attempt to define all the cell populations constituting multicellular organisms, Laszlo G. Lajtha in 1979 postulated the existence of “transit cells” that were different from SCs. These cells were produced by precursor cell populations and were short lived. The “time of transit*”* was defined by a maturation process limiting their proliferative capacity [11]. He also emphasized that “…amplification which occurs in transit populations originating from stem cells results in stem cells being a minority population…” [11], which predicts that proliferation rates in these transit cells will be, at some point, higher than those in SCs.

Further elaboration of the model implied that TACs were irreversibly converted in differentiated cells effectively amplifying each stem cell division and protecting the genetic pool. In addition, each TAC must have a set number of cell divisions [9, 12, 13]. While looking at cultured cells from a teratoma, Rheinwald and Green found previously unidentified epithelial cells that would grow rapidly but only in the presence of fibroblasts. Since these fibroblasts (3T3 cell line) had the capacity to enhance proliferation in keratinocyte cultures, years later, they were successfully used in the treatment of many burned patients [14]. One of the initial experiments consisted of inoculating single cells, isolated from the epidermis and placed in different culture dishes. Twelve days later, the researchers identified three distinct types of founding cells, named holoclones, paraclones, and meroclones [15].

Holoclones showed better growth potential and were believed to be SCs. Paraclones, in contrast, only grew up to 15 cell generations and were believed to be differentiated cells. Meroclones showed an intermediate growth potential. A key observation was that after subculture transfers, holoclones became meroclones, which were gradually converted to paraclones; therefore, a directional restriction in growth potential was identified [15]. Meroclones were the best candidates to possess an enriched population of TACs. These clonogenic epidermal cultures were ideal for studying the transition from SCs to TACs [16]. Some cellular markers were later identified to help distinguish holoclones (B1 integrin and keratin 14) [17] from paraclones (involucrin) [15]. However, as meroclones consist of an intermediate (transit) population of cells, it has been challenging to identify markers that distinguish them from SCs.

**Figure 1:**
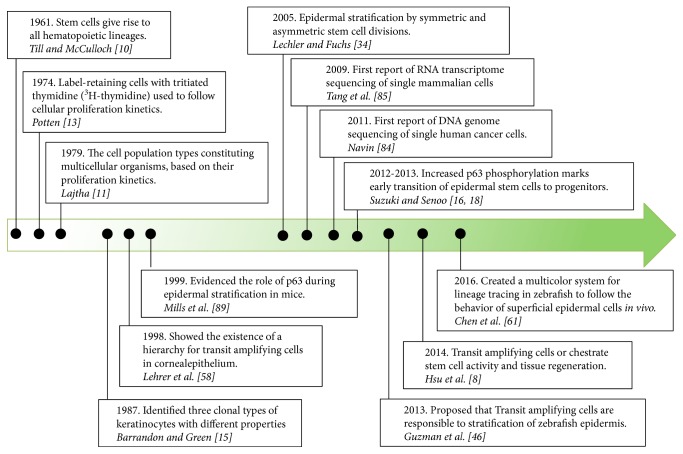
Time line for discoveries that shaped the current concept of Transit-Amplifying Cells.

Epidermal SCs express a transcription factor, known as p63, that was found to be phosphorylated in TACs. Therefore, it has been used to label TACs during regeneration experiments [16, 18]. Currently, most authors recognize that TACs are a subpopulation intermediate between SCs and differentiated cells. Furthermore, TACs have the capacity to amplify the number of terminally differentiated cells that are produced from each stem cell division [19]. It is worth mentioning that some other authors believe that TACs are not part of epidermal homeostasis and tissue formation and renewal [20]. Nonetheless, TACs have been identified in multiple organisms and different organs and tissues [21-28] (see Table 1). We summarize some of the significant contributions that defined the current concept of transit-amplifying cells in Figure 1.

## 3. Gene Expression in Transit-Amplifying Cells: Lessons from Hair Follicles

Essential knowledge about how TACs are regulated come from studies in mammals on the hair follicle (HF). The HF is a particularly useful system in which to study SCs and their progeny since hair growth is fueled by SCs during development, and in adults, the HFs continuously regenerate in alternate cycles of growth (anagen), destruction (catagen), and quiescence (telogen) [29]. Furthermore, mouse HFs are an ideal system to explore possible interactions and regulation between SC and TAC populations. The TACs associated with HFs are located surrounding the dermal papilla (DP) at the base of the HF; these TACs are embedded in matrix cells that enclose the DP. The DP is a specialized niche compartment, and their signals (such as TGFB2 and FGF7) have a strong influence in regulating TAC proliferation, migration, and differentiation. At the same time, TAC signals have a role in SC regulation. For example, the TAC-secreted ligand Sonic Hedgehog (SHH) induces quiescent SCs at the HF bulge, to proliferate [8], hence promoting the anagen hair growth phase.

With the intention to describe a gene expression profile from the HF, a microarray experiment was performed in 2005, where many DP-enriched genes were identified. However, it was not possible to distinguish TAC-expressed genes from those active in other HF cell types [30]. Ten years later and taking advantage of six cell-specific transgenic reporters, it was possible to separate different cell populations in HFs during developmental stages and to perform transcriptomic analysis via next-generation sequencing [31]. More than two hundred genes were expressed by TACs, such as HOXC13, KITL, LEF1, BMP2, WNT10b, FOXP1, and GLI3, among many others (the complete list is found in http://hair-gel.net). Several of these genes are expressed in adult HF-TACs during the anagen growth phase, suggesting that there are similarities between hair growth during development and active HF proliferation cycles in mature organisms.

From the transcriptomic analysis of HF cells (outer root sheath cells, matrix cells, melanocytes, dermal fibroblasts, epidermal cells, TACs, and SCs), it was possible to build an interactome for HF signals that induce development. While epidermal populations express WNT ligands, DP cells express WNT regulators. BMP-signaling molecules are present in all HF cell types, but TACs show high expression of a TGFB pathway negative regulator called BAMBI (BMP and activin membrane-bound inhibitor). NOTCH ligands are expressed by epidermal cells, while downstream effectors of NOTCH are expressed in matrix cells and TACs. One of the conclusions of this transcriptomic analysis is that TACs are significantly involved in signaling and regulation. For example, they interact with melanocytes, outer root sheath cells, and DP cells via the KIT, EFNB2, and SHH signaling pathways, respectively [31]. Furthermore, TACs are in close communication with the DP, actively promoting TAC proliferation, with the result of efficient tissue growth. These DP-TAC interactions may modulate cell cycle frequency in primed SCs, quiescent SCs, and TACs at the HF [8].

A different approach for analyzing dynamic gene expression in TACs was to study histone modifications in adult HFs by chromatin immunoprecipitation and deep sequencing (ChiP-seq). Here, several genes were identified to be expressed in SCs but at the same time repressed in TACs [32]. This gene repression profile, in the form of H3K27-trimethylation, was mediated by Polycomb-group (PcG) proteins over genes such as SOX9, HOXA7, WNT5b, FGF18, and LGR5. At the same time, a different subset of genes, usually PcG repressed in SCs, was becoming activated in TAC progenitors, such as LEF1, BMP4, RUNX2, and WNT5a [32]. Therefore, the transition from SCs to TACs involves PcG-mediated repression of certain genes but activation of some others. The mechanism of switching from a pluripotent state to a differentiated state may be better understood by comparing the functions of activated or repressed genes during this transition.

## 4. Asymmetric Cell Divisions in the Transition from Stem Cells to Transit-Amplifying Cells

Epidermis formation during development is a complex process. Lateral expansion occurs while the organism grows, and at the same time, more epidermal layers are added (stratification). As we mentioned before, SCs may divide by either symmetric cell division (SCD) or asymmetric cell division (ACD). The epidermal basal layer is formed by SCs that divide asymmetrically to produce one daughter cell, which remains at the basal layer, and another daughter TAC that will rapidly proliferate at suprabasal layers. Because of ACD, the number of SCs at the basal layer is kept constant, while the number of TACs rapidly increases, producing new epidermal layers during development [6, 33] or tissue repair [16, 18]. When stratification was initiated during mouse development, an active ACD process could be observed, since over 70% of the mitotic spindles, at the basal epidermal layer, were in a perpendicular orientation, to the basement membrane [34].

Many proteins take part in mitotic spindle reorientation during epidermal stratification by ACD. MINSC is the mouse homolog of the gene *Inscuteable* from *Drosophila melanogaster* that has been implicated in tethering apical multiprotein complexes involved in cell polarization [35]. For instance, overexpression of MINSC increased the frequency of cells with ACD [33]. One of these oligomeric complexes is formed by MINSC, PAR3 (*Partitioning defective-3*), and LGN (homolog of *Pins* from *D. melanogaster*). Once the MINSC-PAR3-LGN complex has been assembled, it recruits NuMA (*Nuclear Mitotic Apparatus*) to the apical cell cortex. The uncoupling of NuMA from MINSC-PAR3-LGN produces more cells by SCD [33]. LGN N-terminal-*TPR* repeats, and C-terminal-*Goloco* domains are essential to recruit NuMA to the complex and its apical accumulation [36]. While the *Goloco* domains bind heterotrimeric G proteins, the *TPR* domain facilitates the interaction between NuMA and MINSC [37, 38]. These multimeric complexes also recruit molecular motors, like dynein and dynactin, needed to shift the mitotic spindle orientation (Table 2) [39].

The reorientation of the mitotic spindle during ACD not only will direct the position of daughter cells but also affects the localization of cell fate determinants as well [40]. This include many cellular components, such as membrane proteins, tight junctions, and organelles that are reorganized [41]. For example, EGFR (epidermal growth factor receptor) was asymmetrically distributed after epidermal ACD [41, 42], which is particularly relevant since EGF is known to control proliferation in epidermal cells. EGFR regulates the NOTCH signaling pathway [43], explaining why blocking ACD impairs NOTCH function [44]. EGFR is also required for TACs to differentiate since it promotes keratin 5/14-expressing epidermal cells to change their expression to keratin 1/10 [45]. Control of cell division rates may also be related to ACD. During zebrafish epidermis development, higher cell proliferation rates were observed to switch from basal cells to suprabasal cells, during stratification [46]. It is clear that molecular mechanisms controlling ACD are essential for the transition from SCs to TACs during epidermis formation [41].

## 5. Cell Proliferation Differences between Stem Cells and Transit-Amplifying Cells

Early on, it was observed that TACs were ephemeral; they divided faster and differentiated rapidly, while SCs were almost nonproliferative in steady-state conditions [11, 15]. Indeed, SCs from adult organisms replicate very slow, as in mice where hematopoietic SCs (HSCs) replicate once every 2.5 weeks [47] or in humans where HSCs replicate every 10 months [48]. During development, SCs proliferate faster while serving as the source of cells for new organs and tissues. However, as organisms mature, SCs divide less and less, moving towards a quiescent state, which may be related to avoiding premature exhaustion and minimizing mutational events [5]. This behavior is critical for therapeutic cell transplants since low proliferation rates may limit tissue repair. In contrast, TACs are highly proliferative making cell cycle dynamics one of the main differences between SCs and TACs.

Cell proliferation rates are directly related to the duration of the G1 cell cycle phase. It is known that cyclin-dependent kinases (CDKs) are sequentially activated or repressed, modulating the duration of G1 phase. Therefore, increasing proliferation rates may involve changes in CDK expression. For example, CDK4, CDK6, and cyclins D1–3 are activated to progress from the G1 to the S phase. In the mouse pituitary gland, it was observed that TACs, but not SCs, express CDK4, suggesting that CDK4 may induce proliferation during the transition from SCs to TACs (Table 2) [49]. Opposite to the activity of cyclins and CDKs, CDKIs (cyclin-dependent kinase inhibitors) such as P21 and P27 are cell cycle regulators that inhibit the function of CDKs and lengthen G1. In P21 and P27 knockout (KO) mice, hematopoietic SCs became depleted, while there was an increase in the number of hematopoietic TACs [50, 51]. This supports the idea that after SC division, P21 is downregulated in the new TAC progenitor and CDK4 expression is activated, resulting in higher proliferation levels.

Cell cycle regulation by P21 is partially mediated by the RB protein (also known as retinoblastoma). Active cell proliferation requires that CDK2 and CDK4 phosphorylate RB (producing its inactive form). In contrast, when P21 inhibits CDK2 and CDK4, RB becomes dephosphorylated (producing the active form), and the cells are switched to a nonproliferative state. It has been observed that overexpression of P21 in cultured mammalian cells induced the depletion of the RB pool [52]. In conditional KO mice for RB, there was a substantial reduction of basal epidermal SCs, in combination with an increased cell proliferation at the suprabasal layers. At the same time, these RB-null mice showed a notorious epidermal thickening [53]. In this work, it was concluded that “RB is essential for the maintenance of the postmitotic state of terminally differentiated keratinocytes, preventing cell cycle re-entry” [53]. Since suprabasal epidermal layers in zebrafish larvae were proposed to contain TAC progenitors [46], it is possible that P21-mediated RB inactivation may promote higher levels of cell proliferation in zebrafish epidermal TACs (Table 2).

Paired-like homeodomain transcription factor 2 (PITX2) promotes keratinocyte differentiation in the skin. PITX2 overexpression activates P21 expression in cultured keratinocytes [54]. Furthermore, microinjection of human PITX2 in zebrafish embryos thickens the epidermis and increases keratinocyte differentiation [54]. The cell cycle could be regulated at different levels. For example, the microRNA *let-7b* is a known cell cycle inhibitor [55] that is present in low amounts in epidermal SCs with high proliferation rates. When these proliferative SCs were transfected with a recombinant lentivirus expressing *miR-let-7b*, cyclin D1 and CDK4 levels were reduced and cell proliferation was halted [56]. In conclusion, the regulation of cellular division rates is a key step in the transition from SCs to TACs and later to differentiated cells.

## 6. The Role of Cellular Migration in the Epidermis

TACs are essential to form new layers of cells by epidermal stratification during development or in regeneration after injury [41, 46, 57]. Consequently, changes in TAC regulation could have dramatic effects on the final thickness, permeability, and appearance of the skin. For example, an increment in the number of TACs may play a role in some cellular hyperproliferative diseases, such as psoriasis [58]. Since signals from the environment may regulate the cellular behaviors of SCs and TACs [31, 59], their tissue localization could be equally important; therefore, cellular migration may also be a key aspect to consider. As we mentioned before, it has been observed, in many animal models, that SCs remain at the basal epidermal layer, while TACs are located at the basal and suprabasal layers [16, 18, 46]. Richardson et al. [60] have recently showed that reepithelialization requires long-range epithelial rearrangements, involving radial intercalations of flattened and elongated cells. Such rearrangements lead to a massive recruitment of keratinocytes from the adjacent epidermis and make reepithelization partially independent of keratinocyte proliferation [60].

A recently created zebrafish transgenic line called “skinbow” is useful for studying epidermal cell movements in an adult fish skin [61]. Using Brainbow-based multicolor, they genetically labeled each cell at the most superficial epidermal layers, also known as “superficial epithelial cells” (SECs). Under homeostatic conditions, epidermal cell replacement in SECs occurred mainly by rearrangement of neighboring cells (78%), rather than by repopulation with newly formed cells. At this point, the authors produced epidermal injury by exfoliation (rubbing dry tissue paper over the skin). Then, they observed the contribution of cell migration from old cells in combination with reepithelization by newly formed keratinocytes. SC activity was monitored by crossing the skinbow line with an FUCCI-based sensor fish line [62]. The epidermis regenerated first by shedding large amounts of SECs and by recruiting preexisting SECs from proximal areas to the wound. After that, there was a cell proliferation burst producing new SECs that became integrated with old SECs. They concluded that the epidermis responds rapidly when suffering from injury by promoting the movement of neighboring cells to protect the remaining tissue while activating proliferation in progenitor cells [61]. In the mammalian epidermis, it is known that proliferation of TACs is activated during regeneration [16, 18, 63]; it is possible that the same is true for zebrafish.

## 7. Transit-Amplifying Cells and Differentiation

Transit-amplifying cells possess the capacity to rapidly amplify the pool of differentiated cells produced at each stem cell division [19]. Currently, it is not known if each TAC is committed to differentiate along one specific lineage or various lineages. In other words, their degree of pluripotency is unknown. Early on, it was suggested that TACs could be precursors of “transit populations” with different fates [11]. In the case of the hematopoietic lineage, two different populations of TACs coexist [64]. TACs from the hematopoietic system are derived from bone marrow, and their proliferation depends on “colony-stimulating factors,” such as erythropoietin, to produce the erythroid lineage. Hematopoietic TACs could be divided into CFC-E (colony-forming cells) and BFC-E (erythrocyte burst-forming cells). CFC-Es require erythropoietin, for survival and proliferation while BFC-Es do not; and in the absence of erythropoietin, only CFC-Es undergo apoptosis whereas the BFC-E population remains [64].

In a different tissue, the mammalian cornea, some experimental data have suggested the existence of two distinct populations of TACs: one at the peripheral cornea and another one at the central cornea. Periphery TACs underwent two rounds of division before becoming postmitotic, while central cornea TACs only required one round of cell division. Then after cornea-induced lessons, one of the TACs remained dormant, while the other was active during reepithelization [58]. Many tissues possess two different populations of SCs, a quiescent population (qSCs) and a primed population (pSCs) that is more sensitive to activation. In the hair follicle, pSCs divides and produces TACs that express SHH that induces qSCs to proliferate [8]. If there are multiple types of SCs and different types of TACs in the same tissue, it could be important to generate a selection protocol to isolate the more proliferative progenitor cells in a tissue destined for cell-based transplants.

## 8. Cell-Based Transplants in the Epidermis for Therapeutic Uses

Chronic diseases, injury, cancer, and birth defects are the leading causes of organ malfunction and tissue disruption. The skin is not an exception, and because it has a prominent role as a protective barrier and in aesthetics, skin injury or chronic disease could have great impacts on a person's physical and physiological well-being. According to the CDC (Centers for Disease Control and Prevention), in 2014, close to 400,000 injuries caused by burning were reported in the US alone (www.cdc.gov). For many years now, skin graft transplants have been the lead option to help patients and heal an injured skin [65]. Skin grafting using autologous tissue is currently used to achieve partial or complete healing in acute or chronic wounds. However, one of the biggest problems in this procedure is the damage produced at the donor site. Therefore, among the things to consider is the success in reepithelialization from the grafted epidermis, the capacity of the donor site to heal, and the time required for complete healing.

To avoid damage to the donor site, in therapeutic skin grafting, it is ideal to obtain as few donor skin cells as possible for them to be used to cover larger portions of the skin. For that reason, epidermal cells are often cultured for 1 to 3 weeks and then used for treating both the wound and the donor site [14, 66]. These cells are usually cultured over commercially available matrices with collagen or with synthetic fibers used for meshing since this method helps to cover larger areas of an injured tissue. Sometimes, epidermal cells are cocultured with dermal cells [67]. Unfortunately, epidermal cultures take too long to proliferate, and after implantation, there are long-term durability issues and severe pain at the skin graft, especially when large surface areas of the body need to be restored [66]. It is worth mentioning that recent advances using epidermal cell suspensions seeded or sprayed at the wound site have shown comparable results to skin grafts [68].

Using SCs as a source for new cells, to restore damaged organs to their original condition is currently the top priority for many researchers and institutions [69-71]. Perfecting their use in cell-based therapies could close the gap towards effective wound healing or improving the quality of life in chronic disease [72, 73]. For example, mesenchymal stem cells (MSCs) can proliferate and differentiate when transplanted to different organs [74]. Notably, when MSCs were placed at the epidermis, they differentiated into epidermal cells [75]. MSCs in skin wounds induce dermal fibroblasts to respond to injury [76]; MSCs also promote vascularization [77] and improve skin and appendage regeneration, such as hair follicles or sweat glands [78]. Therefore, in some cases, MSCs could be a better option than skin grafts that do not regenerate skin appendages. Currently, there are many clinical trials to use MSCs to treat several disorders [74], but none are related to skin injuries or diseases. It is worth mentioning that adipose SCs have similar properties as MSCs in wound repair [79].

The regenerative capacity of the epidermis is related to its high cell division rates; the proliferative cells in the epidermis consist mainly of TACs [8, 16, 18]. In theory, it would be possible to enrich the population of TACs obtained from an epidermal sample using the phosphorylated form of the transcription factor P63 [16, 18] and distinguish them from SCs based on the decreased expression of B1 integrin [17]. It is compelling to see many possibilities for future therapies in skin injury and disease treatment that could be derived from current research from SCs and transit-amplifying cells.

## 9. Future Directions

Bioinformatics tools have been used in the SC field of biology, such as transcriptomic analysis or RNAseq. These data have been used to describe the molecular identity of progenitor cells and to identify new marker genes [80]. Then, these marker genes could be used to label specific SC or TAC subpopulations, which can then be identified or even separated from all other cellular types. Single-cell sequencing (S-CS) [81-83] is a powerful new tool for investigating cellular diversity in several fields of biology, including cancer research. It will be of interest to understand how different cells are involved in tumor progression. Furthermore, it could have far-reaching applications in resolving intratumor heterogeneity, investigating clonality in primary tumors, tracking metastatic dissemination, deciphering the mutation rates and mutation phenotypes, and even understanding resistance to therapy [84]. S-CS has been combined with other approaches, such as cell lineage tracing, or knockout-knockdown strategies. With this interdisciplinary approach, the genotype-to-phenotype relationship was better understood for critical cancer-related genes, such as BRAF (B-rapidly accelerated fibrosarcoma), KRAS (K-RAS oncogene), P53 (P53 transcription factor), and EGFR (epidermal growth factor receptor), with relevance in cancer therapy studies [85].

RNA-seq by S-CS in combination with bioinformatics tools has also been used in developmental biology to better understand the origins of different cell types and how different cell types reach their final identity. For example, Satija et al. used reference datasets, obtained from S-CS RNA-seq profiles, to build spatial in situ gene expression patterns. With this tool in hand, they could predict the expression patterns of multiple genes during zebrafish development [86]. S-CS has also been used to identify new cell types in adult tissues; as an example, a rare type of intestinal cell was recently found. This new cell type is located at intestinal crypts, in mice, and possesses a hormone-secreting function [87]. Selected from the RNA-seq dataset, REG4 could now be used as a biomarker for this new enteroendocrine cell type that is formed by several subpopulations (Table 2). The authors suggested that one of these cell subtypes possesses a TAC-like expression profile [87].

There are several proteins that express in epidermal SCs, but not in TACs, such as B1-integrin, the NOTCH receptor DELTA1, and MCSP (melanoma chondroitin sulphate proteoglycan) (Table 2) [88, 89]. SC-S profiling was performed from single epidermal cells obtained from a human keratinocyte culture [90]. From this experiment, it was possible to detect that SCs have a high expression of LRIG1 (leucin-rich repeats and Ig-like domains 1), which is an EGF receptor antagonist. LRIG1 levels are downregulated for TAC proliferation and further differentiation to occur. SC and TAC researchers, working in different animal models, could use these markers to label and follow SC and TAC dynamics and to identify more specific markers for these essential but ephemeral cell types. One must assume that each cell has a unique transcriptome and possesses intrinsic variations that define its identity, during cell differentiation. Transcriptomic profiling offers a glimpse into the nature of cells in homeostasis and disease, including tumor cells. A better knowledge of the heterogeneity among cells, in the same tissue, would be essential in understanding the role of each cell during development or in any physiological condition [84, 91]. We have emphasized, through this review, that TAC plasticity and proliferative capacity make them worthy for exploration, offering open venues as new possibilities in cell-based transplants for therapeutic use.

## Figures and Tables

**Table 1:** 

Organ or tissue where TACs have been described	References
The cornea in human and mice	[26, 58]
The human mammary gland	[23]
The prostate epithelia	[27]
The mammalian epidermis	[15, 19]
The gastrointestinal tract	[28]
The skin epidermis in zebrafish	[46]
The different types of hair follicles in mammals	[25, 59]
The testis in mammals or male germline in *Drosophila*	[21, 22, 24]

**Table 2:** 

Genes expressed in SCs	Genes expressed in TACs	Organ or tissue	Putative TAC function	References
GFR*α*1+	GFR*α*1−/Miwi2+/Ngn3+	Testis	Represent a novel subpopulation of undifferentiated spermatogonium. Also involved in TAC pluripotency	[24]

*β*1 integrins^Hi^/*α*6 integrin^Hi^/CD71^Low^/delta1^Hi^/desmoglein 3^Low^/EGFR1^Low^/Lrig1^Hi^ MCSP+/delta1+	*β*1 integrins^Low^ MCSP−/delta1−	Epidermal cells	May represent a new population without any characterized function	[88–90]

Reg4	Reg4/ribosomal genes	Intestinal epithelium	TAC populations migrating upward along the intestinal crypt-villus axis	[87]

*β*1 integrin^Hi^/keratin14 p63 ^Hi^/Pp63^Low^	*β*1 integrin^Low^/p63 ^Low^/Pp63^Hi^	Clonogenic cultures and keratinocytes cultures	Increased p63 phosphorylation marks the exit from SC state and could be used to detect epidermal cell stratification	[16, 18]

Gas1+ in Bu-SCs/SHH−	Gas1−/SHH+	Hair follicle in mammals	TACs act as a signaling center between Bu-SCs and DP promoting their proliferation. TACs integrate the timing and frequency for two populations of SCs	[59]

p63/PCNA in basal cells	p63/PCNA in suprabasal cells	Zebrafish epidermis	A proliferation shift from basal to suprabasal cells marks the stratification process	[46]

Jak-STAT signaling		*Drosophila* spermatogonium	The ability of TACs to respond to signals from the SC niche and dedifferentiate into SCs	[22]

Symmetric cell division (SCD) MINSC-PAR3- and LGN- uncoupling of NuMA	Asymmetric cell division (ACD) MINSC^Hi^, PAR3^Hi^, LGN^Hi^ in complex with NuMA	Epidermis stratification	Asymmetric division is essential for TACs formation	[33–39]

p21^Hi^/p27^Hi^/RB dephosphorylated (active form)	CDK2^Hi^ CDK4^Hi^/p21^Low^/RB phosphorylated^Down^ (inactive form)	Mouse pituitary gland and hematopoietic cells	Events that may trigger TAC cell proliferation	[49–51]
